# Editorial: Global excellence in cost and resource allocation: Africa

**DOI:** 10.3389/frhs.2024.1446644

**Published:** 2024-08-16

**Authors:** Olufunke A. Alaba, Goodness Aye

**Affiliations:** ^1^ Health Economics Unit, School of Public Health, University of Cape Town, Cape Town, South Africa; ^2^ Department of Economics, University of Pretoria, Pretoria, South Africa

**Keywords:** cost, efficiency, resource allocation, healthcare utilization, health insurance

**Editorial on the Research Topic**
Global excellence in cost and resource allocation: Africa

The nexus between health and economics is critically important, especially in regions where resources are scarce and the burden of disease is high. Our special journal collection, “Global Excellence in Cost and Resource Allocation: Africa,” aims to shed light on various facets of healthcare management across the African continent. The collection presents rigorous analyses and insightful findings that collectively advance our understanding of how to optimize healthcare resources to improve outcomes for populations in need.

One of the key articles in this collection, “The effect of health insurance coverage on antenatal care utilizations in Ethiopia: evidence from a national survey” (Merga et al.), explores the significant impact of health insurance on the utilization of antenatal care services. The study utilized data from the 2019 Ethiopia Mini Demographic and Health Survey (EMDHS) and included a weighted sample of 3,919 women who gave birth in the last five years. The authors found that women with health insurance coverage were 33% more likely to use antenatal care than those without coverage. Additionally, factors such as age, media access, marital status, education status, wealth index, and economic region were significantly associated with antenatal care utilization. This study highlights the critical role of health insurance, economic empowerment, and education in improving maternal health service uptake in Ethiopia.

In urban settings, access to healthcare is often hindered by infrastructural challenges. The article “Impact of traffic congestion on spatial access to healthcare services in Nairobi” (Mutono et al.) delves into how traffic congestion adversely affects the ability of residents to access healthcare facilities. This research mapped 944 primary, 94 secondary, and four tertiary healthcare facilities in Nairobi County and used traffic probe data to analyze access times during peak and off-peak hours. The study found that during peak hours, less than 70% of Nairobi's 4.1 million population was within a 30 min drive from a health facility, compared to over 75% during off-peak hours. The findings advocate for multisectoral collaborations between urban planners, the health sector, and policymakers to optimize healthcare access for city residents.

Financial efficiency and effective resource allocation are vital for sustainable healthcare systems. The article “Cost-Efficiency Analysis of the Improved Web-Based Planning, Budgeting, and Reporting System (PlanRep) in Tanzania” (Ruhago et al.) examines the cost-efficiency of an innovative web-based system used for planning, budgeting, and reporting in Tanzania. The study found a 53% reduction in total costs incurred by local government authorities (LGAs) for planning and budgeting after introducing the Web-based PlanRep system, dropping from USD 3.8 million in 2017/18 to USD 1.8 million in 2018/19. Additionally, the average time required for these processes decreased from 87 days to only 8 days. The study demonstrates that digital solutions can streamline administrative processes, reduce costs, and enhance transparency and accountability in the health sector.

Financial barriers to healthcare can lead to catastrophic out-of-pocket expenditures, pushing households into poverty. The study “An Analysis of Catastrophic Out-of-Pocket Health Expenditures in Ghana” (Sataru et al.) investigates the extent and determinants of catastrophic health expenditures in Ghana. Analyzing data from the Ghana Living Standards Survey, the study found that as the threshold for catastrophic payments increased from 10% to 25% of total household consumption, the incidence dropped from 1.0% to 0.1%. At the 40% threshold of non-food consumption, the incidence was 0.2%. The study highlighted significant inequalities, with the poorest households experiencing a disproportionately higher risk of financial catastrophe. The findings suggest the need for better-targeted financial risk protection measures to reduce the vulnerability of the poorest households.

In a broader context, these findings resonate with the growing body of literature on healthcare access and equity in Africa. For instance, studies have shown that improving health insurance coverage significantly enhances healthcare utilization and reduces out-of-pocket expenditures, as seen in Ghana's national health insurance scheme (1). Similarly, the impact of infrastructural challenges on healthcare access has been documented, with evidence suggesting that efficient transportation systems can drastically improve health outcomes (2).

Moreover, integrating digital health solutions, such as the PlanRep system in Tanzania, aligns with global trends toward digitalization in healthcare management, which has improved efficiency and accountability in various settings (3). Finally, addressing financial barriers to healthcare is crucial for achieving universal health coverage, as catastrophic health expenditures remain a significant barrier for many households in low- and middle-income countries (4).

These articles provide a comprehensive overview of the challenges and opportunities in healthcare cost and resource allocation across various African contexts. They emphasize the importance of health insurance, efficient urban planning, digital innovations in health administration, and protective measures against catastrophic health expenditures. These studies not only contribute valuable knowledge to the field but also offer practical insights for policymakers, health practitioners, and researchers committed to improving health outcomes in Africa.

The importance of this collection lies not just in the individual findings but in the broader narrative they weave together. The articles collectively advocate for a multifaceted approach to healthcare management that integrates economic, infrastructural, and technological solutions. This holistic perspective is essential for addressing the complex healthcare challenges facing Africa today.

As we continue to explore and address the intricacies of healthcare cost and resource allocation, it is our hope that this collection serves as a catalyst for further research and policy innovation. The insights presented herein are a testament to the potential for achieving global excellence in healthcare through targeted, evidence-based strategies that are tailored to the unique needs of African populations.

We invite readers to engage with these articles, reflect on their implications, and consider how the findings can inform and inspire efforts to enhance healthcare systems in Africa and beyond. Through continued collaboration and commitment to excellence, we can make significant strides toward a future where quality healthcare is accessible and affordable for all.
